# Safety profile of sedative endoscopy including cognitive performance in liver cirrhosis: A double-blind randomized controlled trial

**DOI:** 10.1038/s41598-019-52897-w

**Published:** 2019-11-14

**Authors:** Jeong-Ju Yoo, Hyeon Jeong Goong, Ji Eun Moon, Sang Gyune Kim, Young Seok Kim

**Affiliations:** 1 Division of Gastroenterology and Hepatology, Department of Internal Medicine, Soonchunhyang University school of medicine Bucheon Hospital, Bucheon, Korea; 2 Department of Biostatistics, Clinical Trial Center, Soonchunhyang University Bucheon Hospital, Bucheon, Korea

**Keywords:** Oesophagogastroscopy, Liver cirrhosis

## Abstract

The indiscriminate use of sedative drugs during endoscopy can pose multiple risks including cognitive impairment in advanced liver cirrhosis. However, the data are scarce regarding which sedative drugs are safest in these populations. The aim of this study was to evaluate the safety profiles including cognitive performance among midazolam, propofol, and combination therapy in advanced cirrhotic patients. This double-blind randomized controlled study included 60 consecutive advanced cirrhotic patients who underwent upper gastrointestinal endoscopy. The Stroop application was used to screen for cognitive impairment. Patients were randomly assigned to one of 3 groups, midazolam, propofol, or the combination group, and underwent Stroop test before and two hours after the completion of endoscopy. Hemodynamic safety and the subjective satisfaction score were also evaluated. Patients did not show significant changes in on-time or off-time on the Stroop test before and two hours after sedatives, and there was no significant difference among the 3 treatment groups. Also, there were no significant vital sign changes after sedatives. Time-to-recovery was longest in midazolam group, and patient awakening and patient memory were highest in propofol group. However, all 3 groups showed no difference in patient satisfaction, but the combination group was more preferred in terms of subjective satisfaction by physicians. Factors affecting worsened Stroop speed after sedatives were older age, low education level and high MELD score. All sedative methods using midazolam, propofol, or combination therapy showed similar safety profile in advanced cirrhosis, and were not associated with increased risk of cognitive impairment.

## Introduction

In patients with cirrhosis, the use of sedative drugs results in an increased risks of adverse events due to delayed hepatic clearance^1^. Besides the cardiopulmonary adverse events, acute deterioration of hepatic encephalopathy might occur^2,3^. The spectrum of neurocognitive impairment in liver cirrhosis ranges from unimpaired, minimal hepatic encephalopathy (MHE), to overt hepatic encephalopathy (OHE). Although MHE is a subclinical neurocognitive disorder without clinical symptoms, it is associated with impaired quality of life, employment, driving ability, and progression to OHE^4–6^.

Currently, the most frequently used methods to diagnose cognitive impairment include electroencephalography, critical flicker frequency, Continuous Reaction Time Test, Inhibitory Control Test, and computerized test batteries^7^. However, existing tests for detecting cognitive impairment are time consuming and costly, so they have not been widely used in clinical practice^8^. The Stroop test is one method of detecting hepatic encephalopathy, and has been shown to be an efficacious way to screen for cognitive impairment^9^. A previous study showed that the Stroop smartphone app was a short, valid, and reliable tool for use by cirrhotic patients^10^.

The use of midazolam and propofol in patients with liver cirrhosis has been investigated in a few studies. Although previous reports have suggested the use of propofol rather than midazolam in cirrhotic patients, propofol has no reversal agents and lacks analgesic effects. Recently, a balanced propofol sedation (BPS) method combining the advantages of both drugs has been recommended^11^. However, the safety and efficacy of the combination regimen compared to a propofol alone regimen remains controversial in cirrhotic patients^12,13^. Thus, we investigated the influence of midazolam, propofol, and midazolam plus propofol (BPS) on safety and efficacy during diagnostic upper GI endoscopy, to determine which method can best avoid cognitive impairment, using the Stroop test.

## Results

### Baseline characteristics

Between February 2018 and October 2018, 118 patients were screened and a total of 60 patients were randomized into the study. Flow chart is shown in Supplementary Fig. 1^14^. The baseline characteristics of the patients enrolled in the study are reported in Table 1. The mean age of the patients was 52.63 ± 10.07 years, and 48 patients (80%) were male. When categorized by etiology, 34 patients (56.7%) had alcoholic cirrhosis and 26 patients (43.4%) had non-alcoholic cirrhosis. The median Model for End-Stage Liver Disease (MELD) score was 9.49 points and 37 patients (61.6%) were child-pugh class B or C. Any degree of ascites was observed in 34 patients (56.6%). Esophageal varices were found in 50 patients (83.3%), whereas gastric varices were found in 13 patients (23.7%).

**Table 1:** Baseline characteristics of patients at enrollment.

Characteristics	All (N = 60)	Midazolam (N = 20)	Propofol (N = 20)	Combination (N = 20)	*P*
**Demographics**					
Age (years) – mean ± SD	52.63 ± 10.07	49.75 ± 9.41	53.05 ± 11.48	55.10 ± 8.68	0.237
Sex (male) – number (percent)	48 (80)	15 (75)	17 (85)	16 (80)	0.732
ASA class – number (percent)					0.545
I-II	27 (45)	8 (40)	11 (55)	8 (40)	
III	33 (55)	12 (60)	9 (45)	12 (60)	
Education level – number (percent)					0.287
Low-educated (<9 years)	12 (20)	6 (30)	2 (10)	4 (20)	
High-educated (≥9 years)	48 (80)	14 (70)	18 (90)	16 (80)	
**Etiology** – number (percent)					0.414
Alcohol	34 (56.7)	13 (65.0)	12 (60.0)	9 (45.0)	
Non-alcohol	26 (43.4)	7 (35.0)	8 (40.0)	11 (55.0)	
**Laboratory values** – mean ± SD					
White blood cell count (10^3^/mL)	4.96 ± 2.62	4.81 ± 2.07	5.28 ± 3.17	4.81 ± 2.60	0.808
Hemoglobin (g/dL)	10.8 ± 2.2	10.8 ± 2.5	11.4 ± 1.7	9.9 ± 2.2	0.107
Platelet count (10^3^/mL)	100.0 ± 48.6	97.9 ± 36.4	101.9 ± 64.7	100.3 ± 42.8	0.968
AST (IU/L)	60.0 ± 52.2	74.4 ± 74.6	60.1 ± 36.4	45.6 ± 34.0	0.222
ALT (IU/L)	27.9 ± 27.0	34.3 ± 37.3	27.1 ± 14.5	22.3 ± 24.3	0.379
Total bilirubin (mg/dL)	2.2 ± 2.7	2.5 ± 3.5	2.0 ± 1.9	2.2 ± 2.7	0.839
Serum albumin (g/dL)	3.2 ± 0.7	3.2 ± 0.7	3.2 ± 0.6	3.1 ± 0.6	0.929
Prothromin time (INR)	1.3 ± 0.3	1.3 ± 0.4	1.3 ± 0.2	1.2 ± 0.3	0.940
Serum creatinine (mg/dL)	0.9 ± 0.2	0.9 ± 0.2	0.9 ± 0.1	0.9 ± 0.1	0.823
Serum sodium (mmol/L)	138.5 ± 3.7	138.6 ± 3.8	138.6 ± 3.8	138.4 ± 3.7	0.990
**Liver function**					
Ascites – number (percent)					0.477
None	26 (43.3)	9 (45.0)	11 (55.0)	6 (30.0)	
Mild to moderate	20 (33.3)	7 (35.0)	4 (20.0)	9 (45.0)	
Severe	14 (23.3)	4 (20.0)	5 (25.0)	5 (25.0)	
MELD score – median [IQR]	9.49 [8.19–13.13]	9.36 [8.02–12.71]	10.04 [9.19–13.43]	8.92 [7.45–13.15]	0.608
Child-Pugh class – number (percent)					0.889
Class A	23 (38.4)	6 (30.0)	9 (45.0)	8 (40.0)	
Class B	29 (48.3)	11 (55.0)	9 (45.0)	9 (45.0)	
Class C	8 (13.3)	3 (15.0)	2 (10.0)	3 (15.0)	
**Endoscopic findings** – number (percent)					
Scope retrieval time (min)	11.95 ± 4.85	11.10 ± 4.56	12.45 ± 5.38	12.30 ± 4.72	0.637
Esophageal varices					0.464
No varix	10 (16.7)	2 (10.0)	3 (15.0)	5 (25.0)	
F1	19 (31.7)	7 (35.0)	7 (35.0)	5 (25.0)	
F2	26 (43.3)	11 (55.0)	8 (40.0)	7 (35.0)	
F3	5 (8.3)	0 (0)	2 (10.0)	3 (15.0)	
Gastric varices					0.058
No	47 (78.3)	19 (95.0)	15 (75.0)	13 (65.0)	
Present	13 (23.7)	1 (5.0)	5 (25.0)	7 (35.0)	
**Sedative drug** – median [IQR]					
Midazolam (mg)	2 [0–3]	3 [3–4]	0	1.5 [1–3]	0.002
Propofol (mg)	22.5 [0–45]	0	50 [40–60]	27.5 [20–40]	0.007

Data was reported as median and interquartile range (IQR) presented as median [25th percentile, 75th percentile] or means and standard deviation (SD) (mean ± SD) for continuous variables. Data was reported as frequency (percentage) for categorical variables. Proportions are presented as percentages for categorical variables. P-values were calculated by the Kruskal-Wallis test for continuous variables and chi-square test or Fisher’s exact test for categorical variables.

Abbreviations: SD, standard deviation; ASA class, American Society of Anesthesiologists Classification; AST, aspartate aminotransferase; ALT, alanine aminotransferase; INR, international normalized ratio; MELD, Model for End-Stage Liver Disease; IQR, interquartile range.

### Hemodynamic changes and safety

**Figure 1:**
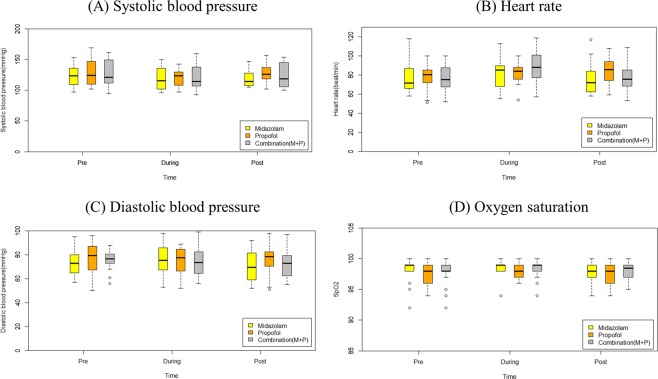
Hemodynamic and pulse oximetry change during upper endoscopy. Hemodynamic change and oxygen saturation using pulse oximetry before, during, and after endoscopy according to each group. (**A**) Systolic blood pressure; (**B**) heart rate; (**C**) diastolic blood pressure; and (**D**) oxygen saturation.

Changes in vital signs before, during, and two hours after endoscopy are recorded in Supplementary Table 1 and Fig. 1. Two hours after endoscopy, systolic blood pressure (SBP) tended to be slightly lower in the M group (114 mmHg) and MP group (119 mmHg), compared to the P group (126 mmHg). However, the differences were not statistically significant between groups (*P* = 0.144; Fig. 1A). When SBP was compared before and after treatment for each drug, all groups showed no significant changes in SBP before or after the procedure. Heart rate (HR), tended to rise during the endoscopy (85 beat/min), but returned to baseline (78 beat/min) after the endoscopy. However, the differences among the groups were not clear (Fig. 1B). Diastolic blood pressure (DBP) and oxygen saturation did not show any significant change before, during, or after the procedure. In addition, there was no significant difference according to groups (Fig. 1C,D). None of the patients underwent major serious adverse events during the endoscopy, and no patients showed paradoxical response after sedative drug injection (Supplementary Table 2).

### Stroop test results before and after sedation

**Figure 2:**
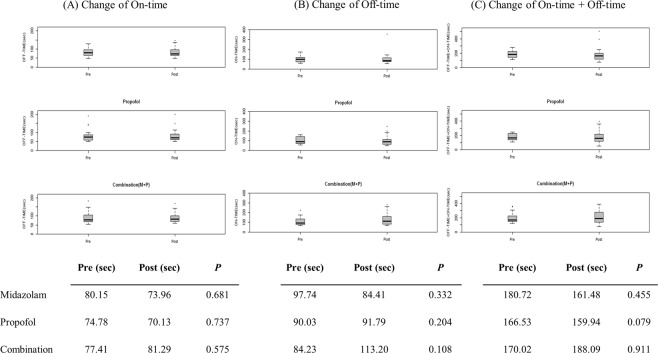
Stroop test change before and after sedation. Change of Stroop test before and after sedation according to each group. (**A**) Change of On-time; (**B**) change of Off-time; and (C) change of Off-time plus On-time.

The total Off-time and On-time from the Stroop test before and two hours after the endoscopy are described in Table 2 and Fig. 2. Median trials for five correct runs on the Stroop application are described in Supplementary Table 3. In all patients, pre-endoscopic Stroop On-time was significantly higher than Off-time (93.5 vs 76.9 seconds; *P* < 0.001), whereas the number of runs needed to complete five correct runs were similar between Off and On states. Two hours after the endoscopy, Off-time was changed from 76.9 to 74.7 seconds (*P* = 0.848) and On-time changed from 93.5 to 92.5 seconds (*P* = 0.457). There were no significant differences between pre-endoscopic and post-endoscopic examinations in the results of Off-time, On-time and Off-time plus On-time (Table 2). Next, we compared the changes in Stroop test results before and after endoscopy for each drug. In the M group, changes of Off-time (Fig. 2A, *P* = 0.681), On-time (Fig. 2B, *P* = 0.332), and Off-time plus On-time (Fig. 2C, *P* = 0.455) were not significantly different after sedation, and these results were similar in the P group (Off-time, *P* = 0.737; On-time, *P* = 0.204; Off-time plus On-time, *P* = 0.079) and the MP group (Off-time, *P* = 0.575; On-time, *P* = 0.108; Off-time plus On-time, *P* = 0.911).

**Table 2:** Time required to complete the Stoop application in the off and on states.

Outcomes	All (N = 60)	Midazolam (N = 20)	Propofol (N = 20)	Combination (N = 20)	*P*
**Off-time (seconds)**					
Pre	76.9 (63.6–96.9)	80.1 (64.4–96.9)	74.7 (57.0–85.8)	77.4 (65.8–106.0)	0.684
Post	74.7 (62.8–98.9)	73.9 (63.8–100.6)	70.1 (59.9–91.0)	81.2 (68.7–101.8)	0.253
Δ Post-Pre	1.6 (−13.8–14.4)	1.6 (−15.0–22.5)	−2.8 (−13.0–13.2)	3.6 (−13.7–15.1)	0.848
**On-time (seconds)**					
Pre	93.5 (74.3–133.0)	97.7 (71.7–123.3)	90.0 (72.2–144.4)	84.2 (78.7–140.4)	0.678
Post	92.5 (77.1–128.2)	84.4 (73.6–118.3)	91.7 (64.8–113.8)	113.3 (80.8–163.4)	0.133
Δ Post-Pre	7.6 (−48.9–12.9)	−15.8 (−57.8–11.5)	−11.8 (−52.5–17.3)	−0.9 (−34.8–21.1)	0.457
**Off-time + On-time (seconds)**					
Pre	172.1 (143.3–226.5)	180.7 (139.2–226.2)	166.5 (137.0–227.5)	170.0 (147.4–227.4)	0.793
Post	166.9 (126.2–231.9)	161.4 (116.0–197.9)	159.9 (116.2–231.5)	188.0 (133.6–285.2)	0.509
Δ Post-Pre	−10.2 (−55.3–18.8)	−10.5 (−51.5–9.7)	−18.9 (−62.5–25.6)	−3.5 (−34.2–28.0)	0.382

Data was reported as median and interquartile range (IQR) for continuous variables. *P* values were calculated by the Kruskal-Wallis test for continuous variables.

### Subjective satisfaction measurement

The time-to-recovery after endoscopy was analyzed. The M group took the longest time (27 minutes) to recover, followed by the MP group (15.0 minutes) and the P group (8.0 minutes). The difference between groups was statistically significant (*P* = 0.006). Next, we evaluated the procedure satisfaction of doctors, nurses, and patients after the endoscopic procedures (Table 3). In the physician group, the MP group showed the highest satisfaction (9.0 points), followed by the M group (8.0 points) and the P group (7.5 points) (*P* = 0.024). Nurses also showed a similar pattern, with higher satisfaction in the order of the MP group (8.5 points), the M group (8.0 points), and the P group (7.0 points), but the results were not statistically significant (*P* = 0.053). Patients were surveyed on four aspects. In patients, overall satisfaction did not show any difference among the groups (*P* = 0.365). Also, the recall of pain or discomfort did not show any significant difference (*P* = 0.127). However, patients who experienced temporary awakening or memory during endoscopy were significantly higher in the P group (both *P* < 0.001).

**Table 3:** Subjective satisfaction measurements.

Outcomes	All (N = 60)	Midazolam (N = 20)	Propofol (N = 20)	Combination (N = 20)	*P*
**Time-to-recovery (minute)**	18.90 ± 13.67	26.80 ± 14.57	12.40 ± 10.52	17.55 ± 12.07	0.006
**Doctors (points/10)**					
Overall satisfaction	8.1 ± 1.3	7.9 ± 1.1	7.6 ± 1.5	8.8 ± 1.0	0.024
**Nurses (points/10)**					
Overall satisfaction	7.7 ± 1.4	7.5 ± 1.3	7.2 ± 1.5	8.3 ± 1.1	0.053
**Patients (points/10)**					
Overall satisfaction	8.6 ± 2.1	8.8 ± 2.4	8.4 ± 2.3	8.8 ± 1.7	0.365
Recall of pain or discomfort	1.5 ± 2.4	0.9 ± 2.1	2.3 ± 2.9	1.2 ± 1.9	0.127
Awakening	3.6 ± 4.1	2.6 ± 3.5	7.0 ± 3.8	1.4 ± 2.9	<0.001
Memory	3.9 ± 4.3	2.6 ± 3.6	6.9 ± 3.8	2.2 ± 4.0	<0.001

Data was reported as means and standard deviation (SD) (mean ± SD) for continuous variables. P-values were calculated by the Kruskal-Wallis test for continuous variables.

### Factors affecting cognitive performance

Finally, we analyzed the factors affecting the cognitive performance using the results of Off-time plus On-time tests after sedation (Table 4). In multivariate analysis, older age [beta coefficients (B) 4.20, standard error (SE) 0.91; *P* < 0.001], low education level (B 85.17, SE 20.99; *P* < 0.001) and high MELD score (B 7.59, SE 1.84; *P* < 0.001) were associated with cognitive impairment showing an increased Off-time plus On-time after sedation. However, sedative drug and groups were not significant factors in univariate and multivariate analyses. Similar results were obtained when the outcome was changed from Off-time plus On-time to On-time (Supplementary Table 4).

**Table 4:** Factors affecting cognitive impairment after sedation using linear regression analysis.

Variable	Univariable		Multivariable	
	B (SE)	*P* value	B (SE)	*P* value
Age	4.29 (1.12)	<0.001	4.20 (0.91)	<0.001
Sex				
Female	1 (Ref)			
Male	1.33 (31.16)	0.966		
ASA class				
I	1 (Ref)		1 (Ref)	
II	72.97 (23.15)	0.003	32.06 (17.43)	0.071
Education level				
High-educated (≥9 years)	1 (Ref)		1 (Ref)	
Low-educated (<9 years)	118.20 (27.02)	<0.001	85.17 (20.99)	<0.001
Etiology				
Non-alcohol	1 (Ref)			
Alcohol	28.46 (24.87)	0.257		
Ascites				
None	1 (Ref)		1 (Ref)	
Mild to moderate	62.43 (26.81)	0.023	−8.17 (21.13)	0.700
Severe	83.10 (29.88)	0.007	31.57 (22.24)	0.162
MELD score	7.53 (2.34)	0.002	7.59 (1.84)	<0.001
Esophageal varices				
No	1 (Ref)			
F1	30.97 (38.04)	0.419		
F2	35.73 (36.23)	0.328		
F3	33.85 (53.33)	0.528		
Gastric varices				
No	1 (Ref)			
Present	−12.97 (30.21)	0.669		
Sedative drug				
Midazolam only	1 (Ref)			
Propofol only	−3.61 (30.52)	0.906		
Midazolam + Propofol	25.07 (0.52)	0.415		

Abbreviations: B, beta coefficients; SE, standard error; ASA class, American Society of Anesthesiologists Classification; MELD, Model for End-Stage Liver Disease.

## Discussion

In terms of the safety of patients with liver cirrhosis, in addition to hemodynamic stability, cognitive impairment must be considered. Although most physicians are aware of the clinical significance of cognitive performance, the tests used to diagnose cognitive impairment, e.g. MHE, so far are expensive, time-consuming, require an expert to conduct, and have not been widely used in real clinical practice^8^. Recently, the Stroop application, a method that can be implemented very simply through a smartphone application, has been developed and compared with other tools (NCT, digit symbol test, block-design test), and verification has been reported. It is easier and simpler than other tests to diagnose cognitive impairment and has proven clinical utility in cirrhotic patients. In our study, even older patients performed a Stroop test with relative ease according to the examiner’s explanation.

To date, there have been three randomized control trials for deterioration of cognitive performance after sedation. One study concluded that there was no difference in the incidence of MHE between midazolam and propofol^15^, and two studies concluded that midazolam increased MHE significantly compared to propofol^16,17^. In our study, deterioration was not observed before and after two hours of sedation in all three groups: midazolam, propofol, and the combination group. We suggest that the difference of the results for each study were because of the following reasons. First, the tests performed to detect cognitive impairment were different in all studies, so it is impossible to directly compare them. Second, the time of follow-up test after sedation was different in each study. We performed the follow-up Stroop test after two hours, in consideration of the half-life of midazolam and discharge time from the recovery room. However, other studies have conducted this test at 30 and 60 minutes, which is likely to result in worse effects for the midazolam group^15,17^. Third, drug doses and protocols tended to differ slightly from study to study. In fact, three studies conducted in Germany, India and Israel used a dose of propofol higher than our study (more than 150 mg *vs*. 50 mg). Asians seemed to reach the sedation level with relatively small doses compared to ethnicities represented in the three studies cited above. Because the body mass index and drug metabolism are different for different races, the protocol dose seems to be different for each race. Therefore, if race is different, direct comparison between studies may be difficult.

In our study, advanced age and deteriorated liver function were associated with worsened cognitive performance after sedatives. Factors reported in other studies were advanced cirrhosis, previous history of OHE^10,18^, and advanced ages^19^, similar to our study. Lower educated patients were also shown at increased risk factor for cognitive impairment in our study, and this is a new discovery not shown by other studies. In other studies, most of the enrolled patients were highly educated persons (median 14 years), and few studies have been conducted on patients with low education levels^10^. Taken together, age and education level should be considered in interpreting the results of the Stroop test.

Our study, unlike other studies, examined the satisfaction scores of medical staff and patients. Despite the fact that subjective satisfaction is a clinically important index, is has not been performed in many studies related to endoscopy in patients with liver cirrhosis. One study reported that patients prefer propofol rather than midazolam, in terms of subjective satisfaction^20^. However, in our study, all patients were satisfied with midazolam, propofol, and the drug combination, and there was no difference among the three groups. Meanwhile, in the medical staff, especially the physician group, the satisfaction of the drug combination was the highest. Patients who experienced temporary awakening were higher in the propofol group, and these items might also affect the satisfaction of the medical staff.

However, our study has the following limitations. First, only a single Stroop test was used as a diagnostic tool of cognitive impairment. Furthermore, exact cut-off for cognitive impairment was not presented. Second, no long-term consequences were evaluated in our patients. Third, this study included only upper GI endoscopy for screening varices, so the result might be different in colonoscopy or other type of endoscopic procedure. Forth, our study only recruited Asian patients and these findings may not be generalized for all different races.

In conclusion, neither midazolam only, propofol only, nor the combination protocol induced significant cognitive function changes in the Stroop test and showed similar safety profile in advanced cirrhosis. All three methods are hemodynamically safe and showed high patient satisfaction, and can be used clinically in patients with advanced cirrhosis.

## Materials and Methods

### Study design and patients

This was a single-center, prospective, double-blind randomized controlled trial at a tertiary referral hospital, performed from February 2018 to October 2018. This trial was registered in Clinical Research information Service (CRIS), which is a member of WHO International Clinical Trials Registry Platform (registration number KCT0002964, date of registration 31/01/2018). Reporting of the study conforms to Consolidated Standards of Reporting Trials (CONSORT) 2010 statement^14^. The inclusion criteria were admitted patients diagnosed with advanced liver cirrhosis between ages 19 and 75, who were to undergo diagnostic upper GI endoscopy to screen for varices. Exclusion criteria were (1) prior history of OHE; (2) evidence of current gastrointestinal bleeding; (3) American Society of Anesthesiologists (ASA) physical classification class IV or higher; (4) use of anti-convulsant drugs within four weeks before endoscopy that could be anticipated to have a reduced sedative effect; (5) patients who were allergic to study drugs, egg, or soybean; (6) patients who were illiterate or color-blind; and (7) patients who refused to participate in the study. The patients were randomly assigned in a 1:1:1 ratio to the midazolam alone group (M group), propofol alone group (P group), or midazolam plus propofol group (MP group) using computer-generated allocation sequences. The randomization of group was managed by a statistician (MJE) not participating in the endoscopic evaluation of the patients and blinded to the investigators and patients.

### Sedation protocol

In the M group, 0.03 mg/kg or 2 mg of midazolam was given to patients aged <65 years or with a body weight >55 kg. In patients with >65 years old or with a body weight <55 kg, the initial dose was reduced by 20 percent. In the P group, 0.5 mg/kg of propofol was given intravenously initially. In patients >65 years old or with a body weight <55 kg, the initial dose was reduced by 50 percent. In the MP group, 0.03 mg/kg or 2 mg of midazolam and 20 mg of propofol were given initially. In patients >65 years old or with a body weight <55 kg, the dose of midazolam was reduced by 20%, and propofol was reduced by 50 percent. After the endoscopy, blood pressure, pulse oximetry, and heart rate were closely observed for all patients in the recovery room. The patients were discharged from the recovery room when the modified Aldrete score was scored at ≤9^21^.

### The assessment of cognitive performance

In this study, all patients underwent the Stroop test to evaluate the change of psychomotor function, which is one of the methods to screen for hepatic encephalopathy^9,10^. We used the Korean Color Word Stroop test (K-CWST, website, http://175.126.38.165), which is a modified version of the Encephalapp Stroop test translated into Korean. A trained nurse performed all tests using an iPAD. All patients underwent the K-CWST before endoscopy and 2 hours after endoscopy.

### Outcome measurements

The primary outcome of this study was the change Stroop test results before and after endoscopy among three sedative protocols. As secondary outcomes, the time to recovery, hemodynamic profiles, and satisfaction scores of patients, nurses, and doctors were analyzed. After two hours, all patients were asked to complete questionnaires to assess overall satisfaction (0, no satisfaction; 10, full satisfaction), recall of pain or discomfort (0, none; 10, extreme), awakening (0, not awakened; 10, fully awakened), and memory (0, no memory; 10 full memory) using visual analogue scale. Also, the endoscopists and the assistant nurses, who were blinded to the chosen protocol, answered the questionnaires about overall satisfaction of the procedures (0, no satisfaction; 10, full satisfaction).

### Sample size calculation

No studies using the Stroop task for evaluation of cognitive performance before and after sedative endoscopy have been reported. Therefore, we referred to a previous study using the number connection test (NCT) for evaluation of MHE before and after sedation with propofol and midazolam^16^. According to their data, the increase in NCT time was −9.5 seconds [95% confidence interval (CI), −15.7 to −4.6] for propofol and 11 seconds (95% CI, −1.2 to 16.1) for midazolam. We estimated an increase in Stroop test time of −9.5 ± 35.8 seconds for propofol and 11.0 ± 19.7 seconds for midazolam after endoscopy. For the combination of propofol and midazolam, we assumed that the increase in Stroop test time would be −2.67 ± 27.8 seconds, using weighted mean of propofol and midazolam. The resulting estimated sample size was 17 patients per group, or a total subject of 51 patients, with an alpha value of 0.05 and power of 90 percent. Considering a 15% drop out rate, 20 patients per group, or a total of 60 patients were required.

### Statistical analysis

To compare the variables between independent groups, a Kruskal-Wallis test was conducted as appropriate. Statistical differences between the groups were investigated using the χ2 test or Fisher’s exact test for categorical variables and the Mann-Whitney U test, or Kruskal-Wallis test for continuous variables. The univariate and multivariate linear regression analysis was also used to find the factors affecting the results of the Stroop test after sedation. A two-tailed p-value of less than 0.05 was considered statistically significant. All statistical analyses were performed using R (version 3.3.3, The R Foundation for Statistical Computing, Vienna, Austria), or SPSS version 21.0 (IBM Corp., Armonk, NY, USA).

### Ethical approval and informed consent

Written informed consents for sedation and endoscopic procedures, and study protocol were obtained from all patients. This study was approved by the institutional review board of Soonchunhyang University Hospital, Bucheon, Korea (SCHBC-2017-12-017-001) and was registered in the Clinical Research Information Service by Korea Centers for Diseases Control and Prevention, Republic of Korea (KCT0002964). Also, all experiments were performed in accordance with relevant guidelines and regulations.

## Supplementary information

Supplementary file

## Data Availability

All authors agreed to make materials, data and associated protocols promptly available to readers without undue qualifications in material transfer agreements.
