# Supplementary material for: Safety profile of sedative endoscopy including cognitive performance in liver cirrhosis: A double-blind randomized controlled trial
Source: Sci Rep. 2019 Nov 14;9:16798. doi: 10.1038/s41598-019-52897-w (PMC6856546; doi:10.1038/s41598-019-52897-w)
Supplement: Supplementary file 1 — Supplementary file [file 41598_2019_52897_MOESM1_ESM.docx]

**Supporting information**

**Safety profile of sedative endoscopy including cognitive performance in liver cirrhosis: A double-blind randomized controlled trial**

**Short title: sedative drugs to avoid cognitive impairment in cirrhosis**

**Authors:** Jeong-Ju Yoo^1*^, Hyeon Jeong Goong^1*^, Ji Eun Moon^2^, Sang Gyune Kim^1^, Young Seok Kim^1^

*^1^Division of Gastroenterology and Hepatology, Department of Internal Medicine, Soonchunhyang University school of medicine Bucheon Hospital, Bucheon, Korea; ^2^Department of Biostatistics,Clinical Trial Center, Soonchunhyang University Bucheon Hospital, Bucheon, Korea*

**Table of Contents**

**Supplementary Figures……………………………………………………………………...…….2**

Supplementary Figure 1. Flowchart showing the flow of participants through the trial[^1^](#_ENREF_1)

**Supplementary Tables……………………………………………………………………...…..….3**

Supplementary Table 1. Vital sign changes during sedative endoscopy

Supplementary Table 2. Adverse events observed during the study

Supplementary Table 3. Median trials for five on correct runs on the Stroop application result

Supplementary Table 4. Factors affecting On-time in the Stroop test after sedation using linear regression analysis

**References ………………………………………….……………………………………...…..….8**

**Supplementary Figure 1. Flowchart showing the flow of participants through the trial** [**^1^**](#_ENREF_1)

**
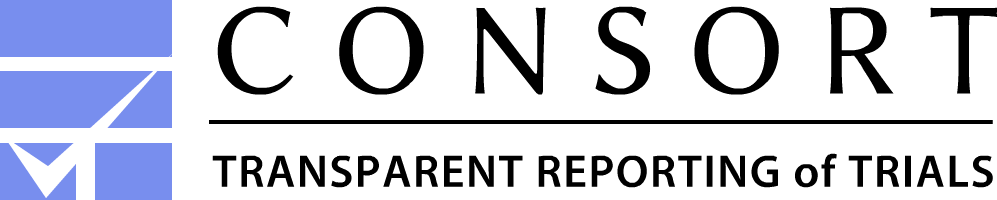
**

## Enrollment

Midazolam alone group

M group

Allocated to intervention (n=20)

♦ Received allocated intervention (n=20)

Lost to follow-up (n=0)

Discontinued intervention (n=0)

## Analysis

## Follow-Up

## Allocation

Randomized (n=60)

Assessed for eligibility (n=118)

**
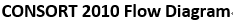
**

Excluded (n=58)

♦ ASA class IV or higher (n=20)

♦ Evidence of gastrointestinal bleeding (n=15)

♦ Prior history of overt encephalopathy (n=14)

♦ Use of anti-convulsant drug (n=3)

♦ Allergic to study drug (n=1)

♦ Illiterate (n=1)

♦ Refuse to participate (n=4)

Midazolam + propofol group

MP group

Analysed (n=20)

Propofol alone group

P group

Analysed (n=20)

Midazolam alone group

M group

Analysed (n=20)

Lost to follow-up (n=0)

Discontinued intervention (n=0)

Lost to follow-up (n=0)

Discontinued intervention (n=0)

Propofol alone group

P group

Allocated to intervention (n=20)

♦ Received allocated intervention (n=20)

Midazolam + propofol group

MP group

Allocated to intervention (n=20)

♦ Received allocated intervention (n=20)

**Supplementary Table 1. Vital sign changes during sedative endoscopy**

| **Outcomes** | **All**  **(N=60)** | **Midazolam**  **(N=20)** | **Propofol**  **(N=20)** | **Combination**  **(N=20)** | ***P*** |
| --- | --- | --- | --- | --- | --- |
| **Systolic blood pressure (mmHg)** |  |  |  |  |  |
| Pre | 122 (110-145) | 123 (109-137) | 124 (109-147) | 121 (111-149) | 0.853 |
| During | 118 (105-134) | 115 (102-136) | 123 (108-130) | 114 (107-138) | 0.824 |
| Post | 121 (109-132) | 114 (106-129) | 126 (117-139) | 119 (106-146) | 0.144 |
| **Diastolic blood pressure (mmHg)** |  |  |  |  |  |
| Pre | 76 (68-82) | 73 (64-81) | 79 (66-87) | 76 (73-80) | 0.478 |
| During | 75 (65-85) | 75 (67-86) | 77 (65-84) | 73 (64-83) | 0.781 |
| Post | 74 (62-80) | 69 (58-82) | 78 (69-82) | 73 (62-79) | 0.257 |
| **Heart rate ( beat/min)** |  |  |  |  |  |
| Pre | 77 (67-86) | 71 (66-88) | 80 (71-85) | 75 (67-87) | 0.622 |
| During | 85 (74-94) | 85 (66-89) | 84 (74-88) | 88 (75-101) | 0.484 |
| Post | 78 (68-88) | 72 (62-85) | 85 (73-95) | 75 (68-85) | 0.065 |
| **Oxygen saturation ( percent)** |  |  |  |  |  |
| Pre | 98 (98-99) | 99 (98-99) | 98 (96-99) | 98 (98-99) | 0.244 |
| During | 99 (98-99) | 99 (98-99) | 98 (97-99) | 99 (98-99) | 0.225 |
| Post | 98 (97-99) | 98 (97-99) | 98 (96-99) | 98 (97-99) | 0.654 |

Data was reported as median and interquartile range (IQR) for continuous variables. P-values were calculated by the Kruskal-Wallis test for continuous variables.

**Supplementary Table 2. Adverse events observed during the study**

| **Adverse event** | **All**  **(N=60)** | **Midazolam**  **(N=20)** | **Propofol**  **(N=20)** | **Combination**  **(N=20)** |
| --- | --- | --- | --- | --- |
| **Overall** | 0 (0) | 0 (0) | 0 (0) | 0 (0) |
| **Respiratory** | 0 (0) | 0 (0) | 0 (0) | 0 (0) |
| Hypoxia (SpO2 <90%) | 0 (0) | 0 (0) | 0 (0) | 0 (0) |
| Upper airway obstruction | 0 (0) | 0 (0) | 0 (0) | 0 (0) |
| **Cardiovascular** | 0 (0) | 0 (0) | 0 (0) | 0 (0) |
| Hypotension | 0 (0) | 0 (0) | 0 (0) | 0 (0) |
| Hypertension | 0 (0) | 0 (0) | 0 (0) | 0 (0) |
| Bradycardia | 0 (0) | 0 (0) | 0 (0) | 0 (0) |
| Arrhythmia | 0 (0) | 0 (0) | 0 (0) | 0 (0) |
| **Procedure-related** | 0 (0) | 0 (0) | 0 (0) | 0 (0) |
| Bleeding | 0 (0) | 0 (0) | 0 (0) | 0 (0) |
| Perforation | 0 (0) | 0 (0) | 0 (0) | 0 (0) |
| Infection | 0 (0) | 0 (0) | 0 (0) | 0 (0) |
| Paradoxical response | 0 (0) | 0 (0) | 0 (0) | 0 (0) |
| Death | 0 (0) | 0 (0) | 0 (0) | 0 (0) |

**Supplementary Table 3. Median trials for five on correct runs on the Stroop application result**

| **Outcomes** | **All**  **(N=60)** | **Midazolam**  **(N=20)** | **Propofol**  **(N=20)** | **Combination**  **(N=20)** | ***P*** |
| --- | --- | --- | --- | --- | --- |
| **Off-state (number)** |  |  |  |  |  |
| Pre | 6.0 (6.0-7.0) | 6 (6.0-7.0) | 6 (6.0-6.5) | 6 (6.0-6.5) | 0.667 |
| Post | 6.0 (6.0-7.0) | 6 (6.0-7.0) | 6 (6.0-6.0) | 6 (6.0-7.0) | 0.392 |
| Δ Post-Pre | 0 (-0.8-0.0) | 0 (-1.0-0) | 0 (-0.8-0.0) | 0 (0.0-0.8) | 0.715 |
| **On-state (number)** |  |  |  |  |  |
| Pre | 6 (6.0-7.0) | 6 (6.0-7.0) | 6.5 (6.0-8.0) | 7 (6.0-7.0) | 0.398 |
| Post | 7 (6.0-8.0) | 6 (6.0-8.0) | 6 (6.0-8.0) | 7 (6.0-9.0) | 0.166 |
| Δ Post-Pre | 0 (-1.0-1.0) | 0 (0-1.8) | 0 (-1.8-0) | 0.5 (0.0-3.0) | 0.049 |

Data was reported as meidan (IQR) for continuous variables. *P* were calculated by Kruskal-Wallis test for continuous variables.

**Supplementary Table 4. Factors affecting On-time in the Stroop test after sedation using linear regression analysis**

| **Variable** | **Univariable** | |  | **Multivariable** | |
| --- | --- | --- | --- | --- | --- |
|  | **B (SE)** | ***P* value** |  | **B (SE)** | ***P* value** |
| Age | 3.10 (0.68) | <0.001 |  | 3.25 (0.59) | <0.001 |
| Sex |  |  |  |  |  |
| Female | 1 (Ref) |  |  |  |  |
| Male | 13.17 (19.62) | 0.505 |  |  |  |
| ASA class |  |  |  |  |  |
| I | 1 (Ref) |  |  | 1 (Ref) |  |
| II | 32.57 (15.25) | 0.037 |  | 6.79 (11.28) | 0.550 |
| Education level |  |  |  |  |  |
| High-educated (≥ 9 years) | 1 (Ref) |  |  | 1 (Ref) |  |
| Low-educated (< 9 years) | 61.99 (17.94) | <0.001 |  | 40.92 (13.58) | 0.004 |
| Etiology |  |  |  |  |  |
| Non-alcohol | 1 (Ref) |  |  |  |  |
| Alcohol | 12.10 (15.82) | 0.447 |  |  |  |
| Ascites |  |  |  |  |  |
| None | 1 (Ref) |  |  | 1 (Ref) |  |
| Mild to moderate | 38.31 (16.72) | 0.026 |  | -5.32 (13.67) | 0.699 |
| Severe | 59.19 (18.63) | 0.002 |  | 29.55 (14.39) | 0.045 |
| MELD score | 4.42 (1.50) | 0.004 |  | 4.83 (1.19) | <0.001 |
| Esophageal varices |  |  |  |  |  |
| No | 1 (Ref) |  |  |  |  |
| F1 | 29.53 (23.90) | 0.222 |  |  |  |
| F2 | 24.39 (22.76) | 0.289 |  |  |  |
| F3 | 12.40 (33.51) | 0.713 |  |  |  |
| Gastric varices |  |  |  |  |  |
| No | 1 (Ref) |  |  |  |  |
| Present | 2.77 (19.12) | 0.885 |  |  |  |
| Sedative drug |  |  |  |  |  |
| Midazolam only | 1 (Ref) |  |  |  |  |
| Propofol only | 1.47 (19.05) | 0.939 |  |  |  |
| Midazolam + Propofol | 27.05 (19.04) | 0.161 |  |  |  |

Abbreviations: B, beta coefficients; SE, standard error; ASA class, American Society of Anesthesiologists Classification; MELD, Model for End-Stage Liver Disease

**References**

1 Schulz, K. F., Altman, D. G., Moher, D. & Group, C. CONSORT 2010 statement: updated guidelines for reporting parallel group randomised trials. *Bmj* **340**, c332, doi:10.1136/bmj.c332 (2010).
